# l-Theanine extends lifespan of adult *Caenorhabditis elegans*

**DOI:** 10.1007/s00394-012-0341-5

**Published:** 2012-03-16

**Authors:** Kim Zarse, Saskia Jabin, Michael Ristow

**Affiliations:** 1 Department of Human Nutrition, Institute of Nutrition, University of Jena, 07743 Jena, Germany; 2 Department of Clinical Nutrition, German Institute of Human Nutrition Potsdam-Rehbrücke, 14558 Nuthetal, Germany

**Keywords:** Amino acids, Aging, Anti-aging, Lifespan, Signaling, Pharmacology, Nutrition, Nutrients, Phytochemicals, Tea, Model organisms, Metazoans, Nematodes, *Caenorhabditis elegans*, Calorie restriction mimetics, Exercise mimetics, Hormesis, Mitohormesis

## Abstract

**Purpose:**

Compounds that delay aging in model organisms may be of significant interest to anti-aging medicine, since these substances potentially provide pharmaceutical approaches to promote healthy lifespan in humans. We here aimed to test whether pharmaceutical concentrations of l-theanine, a putative anti-cancer, anti-obesity, blood pressure-lowering, and neuroprotective compound contained in green tea (*Camellia sinensis*), are capable of extending lifespan in a nematodal model organism for aging processes, the roundworm *Caenorhabditis elegans*.

**Methods:**

Adult *C. elegans* roundworms were maintained on agar plates, were fed *E. coli* strain OP50 bacteria, and l-theanine was applied to agar to test (1) whether it may increase survival upon paraquat exposure and (2) whether it may promote longevity by quantifying survival in the presence and absence of the compound.

**Results:**

l-theanine increases survival of *C. elegans* in the presence of paraquat at a concentration of 1 micromolar. l-theanine extends *C. elegans* lifespan when applied at concentrations of 100 nM, as well as 1 and 10 micromolar.

**Conclusions:**

In the model organism *C. elegans*, l-theanine is capable of promoting paraquat resistance and longevity suggesting that this compound may as well promote healthy lifespan in mammals and possibly humans.

## Introduction

Promotion of longevity and in particular extension of healthy lifespan (also named ‘healthspan’) is of eminent interest to most humans. Specific mutations have been shown to extend the lifespan of model organisms dramatically, while more readily available interventions, including calorie restriction, extend life expectancy of model organisms, however, less strikingly.

Accordingly, considerable effort has been invested to identify naturally occurring and/or pharmaceutical compounds that promote longevity in model organisms. A number of such compounds have been identified in recent years, including rapamycin, 2-deoxy-d-glucose, resveratrol, and additional phytochemicals (as summarized in [1]).

l-theanine is an amino acid known to be contained in green tea (*Camellia sinensis*) and generally considered as safe in humans. l-Theanine has been previously shown to enhance the anti-tumor activity of chemotherapeutic agents [2]. Moreover, l-theanine has been repeatedly shown to promote cognition and mental state and may be counteracting beta-amyloid formation in murine models of Alzheimer’s disease [3, 4]. Last, l-theanine may exert desirable effects on body mass [5] as well as blood pressure [6].

We here have tested whether l-theanine at pharmaceutical doses may be effective in promoting stress resistance and/or extending lifespan of a metazoal model organism, *C. elegans*.

## Methods

### Compounds

l-Theanine was obtained from Sigma-Aldrich (Munich, Germany).

### Maintenance and analyses of nematodes

The *C. elegans* strain used was wild-type N2 (var. Bristol). All experiments were performed exactly as previously described [1, 7–9] except that streptomycin and 5-fluoro-2′-deoxyuridine were omitted, and synchronization was performed as previously described [7]. *E. coli* OP50 bacteria were heat-inactivated as previously described [7] for 45 min and used as the only food source.

### Paraquat stress resistance assay

For paraquat stress resistance assays, L4 larvae were put onto control NGM agar plates and plates containing l-theanine. After 6 days of pre-treatment, about 300 worms of each group were transferred to NGM agar plates containing 10 mM paraquat and survival rate was determined by daily scoring of dead animals.

### Lifespan assay

L4 larvae were put onto NGM agar plates (with and without l-theanine) and worms were transferred daily to fresh plates during the reproduction period. After egg laying stopped, nematodes were transferred every second to third day and scored for dead animals. Animals which died from premature death, e.g. internal hatching were censored.

### Statistical analysis

The log-rank test was carried out to calculate statistical survival distributions between the different groups in the stress assay as well as in the lifespan assays.

## Results

### l-Theanine promotes paraquat resistance in *C. elegans*

**Fig. 1:**
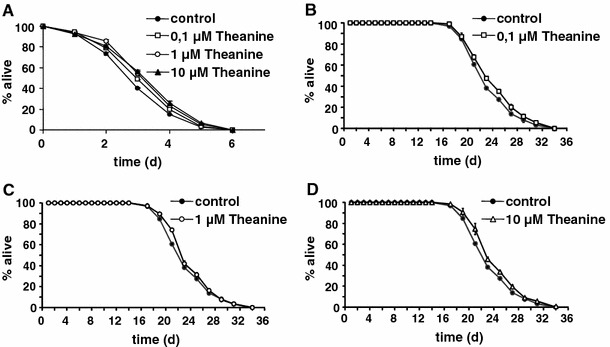
l-Theanine promotes paraquat resistance and extends lifespan of adult *C. elegans*. **a** Depicts relative survival of nematodes on agar plates containing 10 mM paraquat pretreated with l-theanine at three different concentrations. **b**–**d** show three separate lifespan analyses with several hundred nematodes each (see Table 1 for details) at three different concentrations of the compound (100 nM, 1 and 10 micromolar)

To test this possibility we pre-treated adult nematodes for 6 days with l-theanine, and exposed them subsequently to paraquat, a toxic agent known to continuously generate oxidative stress emanating from the mitochondria. At a concentration of 1 micromolar, l-theanine significantly increases paraquat resistance in *C. elegans* (*P* = 0.04*; Fig. 1a). These findings indicated that l-theanine causes increased paraquat resistance in *C. elegans*.

**Table 1:** Life span results and statistical analyses of life span assays

Treatment	Maximum life span (d) 80th percentile (±SD)	Mean life span (d) 50th percentile (±SD)	*P* value (vs. control)	Number of experiments (*n*)	Number of nematodes (*n*)
H_2_O (control)	24.9 ± 0.1	22.1 ± 0.1	n. a.	3	396
0.1 μM l-Theanine	26.0 ± 0.4	22.9 ± 0.2	0.00076***	3	385
1 μM l-Theanine	25.5 ± 0.2	22.8 ± 0.1	0.00858**	3	378
10 μM l-Theanine	25.5 ± 0.6	22.6 ± 0.6	0.00256**	3	365

**P < 0.01, ***P < 0.001

### l-Theanine extends *C. elegans* lifespan

While the following apparently does not apply in all cases [10], increased resistance against paraquat stress suggests that l-theanine may exert effects on *C. elegans* lifespan. Applying this compound at three different concentrations (100 nM, 1 and 10 micromolar) to *C. elegans* using the above-mentioned methods extends lifespan significantly (Fig. 1b–d) (*P* < 0.001***, *P* = 0.008** and *P* = 0.002**, respectively). This effect appears not to be strictly dose-dependent, since no such correlation could be seen for ROS defense capabilities while all concentrations evaluated had a lifespan-extending effect. The maximum observable effect on mean lifespan and maximum lifespan (80th percent percentile) was 0.8 days and 1.1 days, respectively, which occurred at a concentration of 100 nM.

## Discussion

To potentially support the ongoing search for compounds that may promote human health especially at higher age, we here show that l-theanine promotes both stress resistance and longevity in a nematodal model organism, the roundworm *C. elegans*.

We find that l-theanine is extending *C. elegans* lifespan at a wide range of concentration (i.e. from 100 nM up to 10 micromolar) while resistance to 10 mM paraquat was found to be increased at median concentration (1 micromolar) only. The reasons for these differences are unclear and require further investigation.

Whether the effects of the compound on stress resistance and lifespan are related to known anti-oxidant activities of l-theanine [4] remains to be evaluated. Moreover, it will be of interest whether signaling pathways may be involved into the lifespan-promoting effects of l-theanine.

l-Theanine is an amino acid particularly contained in green tea. The compound has previously been shown to exert positive effects on mental state and may prevent an Alzheimer’s disease-like phenotype in murine models of this disease [3, 4]. Moreover, it appears to exert desirable effects on body mass [5] as well as blood pressure [6].

Since the current study has been performed in the model organisms *C. elegans*, it is unclear whether our results can be extrapolated to mammals or even humans. Hence, further studies will have to show whether l-theanine has any effect on mammalian or human health span and/or longevity. However, other compounds that have been identified using a similar, metazoan-based approach have been shown to be effective in rodents [1].

Taken together, these findings indicate that l-theanine extends *C. elegans* lifespan suggesting that this compound may be worth evaluating in mammals and potentially humans in regard to prevention of aging and age-associated diseases.
